# The Kanavel Sign Revisited

**DOI:** 10.31662/jmaj.2024-0199

**Published:** 2024-09-27

**Authors:** Vitorino Modesto dos Santos

**Affiliations:** 1 Armed Forces Hospital, and Catholic University, Brasília-DF, Brazil

**Keywords:** infectious disease, Kanavel sign, pyogenic flexor tenosynovitis

## Dear Editor:

A 72-year-old man exhibiting Kanavel sign due to pyogenic flexor tenosynovitis (PFT) in the left hand, confirmed by magnetic resonance imaging (MRI) revealing fluid collection along the flexor tendons, was recently described in this journal by Takanosu T and Uyama Y ^(1)^. He underwent prompt antibiotic therapy and surgical irrigation debridement successfully. The authors emphasized the earliest diagnostic suspicion index by the Kanavel sign; they also stressed the importance of paying more attention to this condition in daily practice ^(1)^. Therefore, comments on the three cardinal signs of PFT, flexor sheath tenderness, flexed position of the affected digit, and painful passive digital extension, described by Allen B. Kanavel in 1912, in addition to a fourth sign, fusiform swelling of the digit, are useful for accurate diagnosis ^(2), (3), (4), (5)^. Studies using contrast-enhanced computed tomography (CT), magnetic resonance imaging (MRI), and ultrasound (US) contribute to diagnosis, nonoperative management of PFT includes antibiotics, arm elevation, and splinting ^(1), (2), (3), (4), (5)^.

The reviewed data from 39 patients (71.9% males, mean age 9.5 ± 5.5 years revealed that at least 62% patients had 3 Kanavel signs on presentation, 34% had 4 signs, and 3 patients had 0 to 1 sign ^(2)^. Further, a flexed position of the digit was the least common of the signs; the most frequent causative agents were *Staphylococcus aureus* (69%) and *Pasteurella multocida* (13%), followed by infections by multiple organisms (19%). Finally, antibiotic therapy was administered in all cases for 1-16 days ^(2)^. In 81% of cases, the initial antibiotic schedule was effective; methicillin-resistant *S. aureus* was sensitive to vancomycin, trimethoprim-sulfamethoxazole, or clindamycin ^(2)^. All patients underwent incision and drainage (repeated in 18%), surgical incisions were limited (80%), mid axial (13%), or Bruner’s (7%); mean hospitalization duration was 5.1 days ^(2)^. Two groups of 35 adults were compared: one with FTS and another with finger cellulitis (FC), by axial CTs of the coronal and sagittal planes and tendon sheath/tendon width; the tendon sheath/tendon was recorded as a ratio in coronal (CR) and sagittal (SR) planes ^(3)^. More Kanavel signs were found in the FTS (2.9 vs. 0.5) with larger CR and SR; the likelihood of FTS increased (5.9% and 5.5%) for every 0.1 of CR and SR increase. In addition, there was an increase of 14% for every additional Kanavel sign observed ^(3)^. The authors emphasized the value of CT ratios to confirm FTS, either isolated or in combination with Kanavel’s signs, because CR and SR improve the diagnosis of FTS ^(3)^. A retrospective evaluation of the presence or absence of the four Kanavel signs and tenderness over the A1 pulley on the affected digits (or T1 pulley of the thumb) to rule out PFT involved two groups of cases: non-PFT infections (n = 21) and PFT infections (n = 12) ^(4)^. There was a statistically significant difference related to the presence of all Kanavel signs; the sensitivity of the A1 pulley presented a higher odds ratio, positive predictive value, and specificity, in addition to accuracy, when compared with all Kanavel signs ^(4)^. Therefore, the authors concluded that tenderness at the A1 pulley is a useful specification of tenderness over the course of the flexor sheath to aid in the diagnosis of PFT ^(4)^. Therefore, the authors concluded that tenderness at the A1 pulley is a useful specification of tenderness over the flexor sheath course, which may contribute to PFT diagnosis ^(4)^. Radiographic parameters of soft tissue dimensions were compared in adults with finger infections to study differences between cases with PFT and non-PFT infected digits ^(4)^. Another retrospective study on patients with a finger infection and radiographic findings included two groups: PFT (n = 31) and non-PFT infections (n = 31) based on purulence in the tendon sheath, or positive culture growth from the sheath at surgery ^(5)^. PFT was characterized by differential volar soft tissue thickness minus dorsal soft tissue thickness on images at the proximal phalanx level: 9 ± 1 mm vs 5 ± 1 mm for non-PFT. Differences of ≥7 mm had a positive predictive value of 82%, sensitivity of 84%, and specificity of 74%; 10 mm predicted PFT infection with 76% probability ^(5)^. The authors commented on “fusiform swelling” being a misnomer for acute PFT fingers ^(5)^.

Overall, reports of case studies may increase the interest and suspicion index of healthcare workers in uncommon conditions involving frequent diagnostic challenges.

## Article Information

### Conflicts of Interest

None

### Author Contributions

VMS wrote the first manuscript, and edited and approved the final manuscript.

### ORCID iD

https://orcid.org/0000-0002-7033-6074

### Approval by Institutional Review Board (IRB)

IRB approval was not required for this manuscript.

### Informed Consent

Informed consent was not required for this manuscript.
